# miRNAs作为癌症预测和预后标志物的巨大潜能

**DOI:** 10.3779/j.issn.1009-3419.2013.01.12

**Published:** 2013-01-20

**Authors:** William CS CHO, 娟 南

**Affiliations:** 1 Department of Clinical Oncology, Queen Elizabeth Hospital, Hong Kong; 2 天津医科大学总医院，天津市肺癌研究所，天津市肺癌转移与肿瘤微环境重点实验室; 3 香港特别行政区 伊利沙伯医院 临床肿瘤科

**Keywords:** 生物标志物, 癌症, 小分子RNA, miRNA, 预测标志物, 预后标志物

**“因为不同癌症疗法对不同亚型患者有效，所以急需新型预测和预后标志物来提高癌症患者的疗效。”**

miRNAs作为细胞通路的重要调控因子，在癌变过程中起着关键作用。最近研究已鉴定出许多潜在癌症标志物的miRNAs，部分miRNAs起癌基因、抑癌基因或转移调节因子的作用。因为不同癌症疗法对不同亚型患者有效，所以急需新型预测和预后标志物来提高癌症患者的疗效^[1]^。

**miRNAs作为抗癌治疗疗效的分子预测标志物及miRNAs在癌症中的预后价值**

为了改善抗癌治疗患者的预后，亟待发现生物标志物以预测患者抗癌治疗疗效，并帮助研究者设计可更好地分层患者的临床试验。小细胞肺癌肿瘤样本的微阵列分析显示，miR-92a-2^*^、miR-147和miR-574-5p与耐药明显相关。肿瘤中miR-92a-2^*^的高表达与小细胞肺癌患者生存期缩短亦相关。这些miRNAs有可能用于筛查小细胞肺癌患者的再次耐药风险^[2]^。

在胰腺癌术后患者中发现，miR-142-5p高表达的吉西他滨治疗患者明显比低表达者的生存期长。该miRNA可能成为术后胰腺癌患者采用吉西他滨治疗疗效的预测标志物^[3]^。另一方面，基于紫杉醇治疗未完全缓解的卵巢癌患者的miR-200c水平较完全缓解者低。低肿瘤miR-200表达与高β-tubulin Ⅲ蛋白含量明显相关。此miRNA可能成为卵巢癌患者采用基于紫杉醇治疗疗效的生物标志物^[4]^。在单一采用他莫昔芬治疗的雌激素受体阳性的乳腺癌患者中发现，miR-210表达与临床预后不良相关。功能分析显示，miR-210参与细胞增殖、迁移和侵袭^[5]^。

在不同的恶性肿瘤中，异常的miRNA表达可能具有潜在的预后价值。对源自术前未接受任何治疗的Ⅰ期-Ⅲ期鳞癌(squamous cell carcinoma, SCC)患者的配对癌组织和非癌组织的miRNA表达检测发现，miR-31高表达与生存期不佳相关。肿瘤抑癌基因DICER1被鉴定为miR-31的靶标，人肺癌细胞中miR-31的表达可抑制DICER1的活性^[6]^。在食道SCC患者中也发现，血清miR-31高表达者无复发生存期和癌症相关生存期的预后较差。体外研究显示，miR-31可促进食道SCC集落形成、迁移和侵袭。三种肿瘤抑癌基因(*EMP1*、*KSR2*和*RGS4*)被证实为miR-31的靶标^[7]^。该miRNA可能成为SCC的潜在预后生物标志物。

**“癌症研究的一项重要挑战是研发标准治疗后肿瘤复发的可靠预测标志物以决定是否需要立即进行辅助治疗。”**

可切除的胰腺导管腺癌患者的临床协变量独立分析显示，miR-21高表达和miR-34a表达降低与术后总生存期(overall survival, OS)不佳明显相关。这些miRNAs的异常表达水平可能成为胰腺导管腺癌的预后生物标志物^[8]^。癌症研究的一项重要挑战是研发标准治疗后肿瘤复发的可靠预测标志物以决定是否需要立即进行辅助治疗。从福尔马林固定样本中鉴定出两个miRNAs和十个蛋白编码基因(miR-519d、miR-647、*ANXA1*、*BID*、*CCNG2*、*ETV1*、*FBP1*、*LETMD1*、*NOTCH3*、*RAD23B*、*SIM2*和*TNFRSF1A*)，有助于鉴别前列腺癌患者术后有无生化复发。这组标志物可能提高对前列腺癌患者根治性前列腺切除术后预测的准确性^[9]^。

对采用利妥昔单抗、环磷酰胺、阿霉素和长春新碱治疗的弥漫性大B细胞淋巴瘤患者样本进行单因素分析发现，miR-18a表达与OS相关，而miR-222表达与无进展生存期相关。包含国际预后指数的Cox回归分析显示，这些特定miRNAs的表达水平是弥漫性大B细胞淋巴瘤患者生存期的独立预测因子^[10]^。另一方面，miRNA分析显示，侵袭性甲状腺乳头状癌较非侵袭性甲状腺乳头状癌的miR-146b和miR-222上调，而miR-34b和miR-130b下调。在BRAF阳性的肿瘤中，miR-146b与侵袭性甲状腺乳头状癌密切相关。在侵袭性甲状腺乳头状癌中，MET被鉴定为miR-34b的潜在靶基因^[11]^。在胃癌中也发现，miR-146a低表达与淋巴转移、静脉侵袭和OS相关。miR-146a的异常表达可抑制胃癌细胞的侵袭和转移，并下调及靶向*EGFR*和*IRK1*的表达^[12]^。

循环miRNAs是癌症潜在的非侵入性预后生物标志物。在源自早期非小细胞肺癌和匹配对照组患者的血浆和血清配对样本研究中，血浆let-7b表达减少与所有患者的癌症特异性死亡率中等程度相关，血清miR-223表达减少则与IA期/B期患者的癌症特异性死亡率中等程度相关^[13]^。另一方面，在脊髓增生异常综合征患者中，血浆let-7a和miR-16水平是双峰的。它们的表达水平与OS和无进展生存期明显相关。多因素分析显示，let-7a是该人群OS的独立预测因子^[14]^。另一最近研究证实，在鼻咽癌患者中一个包括5个miRNAs的标志物(miR-142-3p、miR-29c、miR- 26a、miR-30e和miR-93)与无疾病生存期明显相关。此生物标志物组可以增加TNM分期系统的预后价值，并有助于为存在进展高风险的患者制定治疗方案^[15]^。

为检测直肠癌是否存在转移，一个包括5个miRNAs (let-7a、miR-21、miR-135a、miR-206和miR-335)表达水平的预后标志物也被鉴定出来。其中，在KRAS突变阳性肿瘤的转移病变中let-7a表达增高。该包括5个miRNAs的标志物可能成为直肠癌的潜在预后工具^[16]^。同样地，在室管膜瘤和正常脑组织中检测miRNAs表达发现，miR-203是预测复发的独立标志物，与OS密切相关的3个miRNAs(let-7d、miR-367和miR-596)也被鉴定出来^[17]^。通过原位杂交筛查let-7家族发现，let-7g表达减少也可成为乳腺癌患者淋巴转移和生存状况差的预后生物标志物。剔除非转移性乳腺癌细胞中let-7g表达导致癌细胞从原位通过*GAB2*和*FN1*以及后续*p44*/*42 MAPK*和特定基质金属蛋白酶的激活而迅速转移^[18]^。

**“……解密miRNAs在复发和转移信号通路中的作用，将有助于更好地利用miRNAs对癌症治疗的预测和预后价值。”**

在乳腺癌病例中，完整的miRNA和mRNA全表达谱已鉴定出与无远处复发生存期独立相关的9个miRNAs (miR-27b、miR-29c、miR-30c、miR-30e-3p、miR-128a、miR-150、miR-210、miR-342和miR-548d)。其中，miR- 27b、miR-150和miR-342在受体三阴性乳腺癌中具有预后价值^[19]^。*MLLT11*是小儿急性髓细胞白血病预后不佳的生物标志物。体外转染实验显示，miR-29b直接调节*MLLT11*的表达。miR-29b低表达和*MLLT11*高表达的急性髓细胞白血病患者的OS不佳，提示miR-29b可能是急性髓细胞白血病患者的潜在预后生物标志物^[20]^。另一方面，甲状腺髓样癌的miRNA谱显示，在甲状腺髓样癌中，miR-183和miR-375的过度表达可预测外侧淋巴结转移。它们与残余病变、远处转移和死亡相关。在人甲状腺髓样癌细胞中，敲除miR-183表达导致活细胞数明显减少和自噬相关蛋白LC3B表达显著增高^[21]^。

## 讨论与展望

探寻可提供有用信息的生物标志物已成为过去十年癌症研究的主要焦点。因为血液易于通过轻微创伤性方式收集，故对于肿瘤生物标志物检测来说血液为理想的样本类型^[22]^。一些研究也曾尝试采用源于粪便的miRNAs作为胃肠癌的标志物^[23, 24]^。采用不同样本类型使得新型标志物的检测受到不同程度的限制。另一方面，未来的趋势是联合多个miRNAs作为标志物组，这通常有助于提高敏感性和特异性。同样地，miRNA标志物与其它分子标志物(如蛋白标志物、SNPs和DNA甲基化标志物)的联合也可能有助于更好地预测个体患者的治疗效果。但是，尽管已报道许多预测和预后标志物具有统计学上的明显差异，但将已有研究成果应用于临床实践仍需严格地验证^[25]^。为了提高生物标志物的可靠性、质量保证和有效性，还需要对不同样本类型制定标准的制备过程并设计更好的方法来评估miRNA标志物的质量。一个稳定可靠的miRNA标志物需经多中心研究共同验证。癌症是一种复杂的疾病，解密miRNAs在复发和转移信号通路中的作用，将有助于更好地利用miRNAs对癌症治疗的预测和预后价值。尽管仍有许多挑战亟待解决，但是最近鼓舞人心的结果显示，miRNAs有成为对人类癌症有用的预测和预后标志物的潜能。

## Financial & competing interests disclosure

*The author has no relevant affiliation or financial involvement with any organization or entity with a financial interest in or financial conflict with the subject matter or materials discussed in the manuscript. Tis includes employment*, *consultancies*, *honoraria*, *stock ownership or options*, *expert testimony*, *grants or patents received or pending*, *or royalties*.

*No writing assistance was utilized in the production of this manuscript*.
